# Does vitamin D status correlate with clinical and biochemical features of polycystic ovarysyndrome in high school girls?

**Published:** 2014

**Authors:** Reza Ghadimi, Sedighe Esmaeilzadeh, Marmar Firoozpour, Asal Ahmadi

**Affiliations:** 1 Social Determinants of Health Research Center, Babol University of Medical Sciences, Babol, Iran.; 2 Fatemeh Zahra Infertility and Reproductive Health Research Center, Babol University of Medical Sciences, Babol, Iran.; 3 Babol University of Medical Sciences, Babol, Iran

**Keywords:** Polycystic ovary syndrome, Vitamin-D, Calcium, Younger girls

## Abstract

***Background: ***Prevalence of polycystic ovary syndrome (PCOs) is increasing particularly among the female adolescents and young women. It has been hypothesized that disturbance in calcium and vitamin-D metabolism may affect the symptoms of this syndrome. This study was designed to investigate the relationship between vitamin-D and calcium with metabolic parameters and other characteristics of the PCOs.

***Methods:*** The study included 192 Iranian girls (16-20 years old), of whom 104 had PCOs and 88 were non-PCOs controls. Serum 25(OH) D and calcium level was measured. Anthropometric components, endocrine, metabolic components and insulin resistance were determined in PCOs subjects.

***Results:*** Mean 25 (OH) D was significantly lower in cases (9.7±4.8) than controls (12.3±11.9) but calcium level did not differ between the two groups (9.3±0.3 vs 9.4±0.4). No significant correlations were found between 25(OH) D levels and lipid profile, FBS, fasting insulin endocrine parameters such as testosterone, free testosterone, FSH, LH, and prolactin.

***Conclusion: ***Although hypovitamionos D was common is PCOs but did not correlate with clinical features or complications of obesity and insulin resistance PCO like severity of syndrome between vitamin-D deficiency and its severity with some features and complications of PCOs including obesity, insulin resistance.

Polycystic ovary syndrome (PCOs) is a prevalent endocrine disorder among women of reproductive age. Clinically, this is characterized by chronic anovulation, insulin resistance, and hyperandrogenic symptoms like oligomenorrhea and hirsutism. The first early signs of PCOs can also be a premature adrenarche with a precocious appearance of pubic hair (1). However, in affected adolescence, oligomenorrhoea / menstrual irregularity may be detected later in life (2, 3). 

Although the symptoms of PCOs begin usually in adolescence, it is believed that its origin lies in childhood or fetal life. In addition, it is not diagnosed until 2-3 years after menarche (4, 5). It is suggested that there is a link between vitamin-D and the pathogenesis of obesity in adults and children (6-9). Vitamin D is thought to influence the development of PCOs through gene transcription (10) and hormonal modulation influences insulin metabolism and fertility regulation, but evidence suggests that vitamin D levels are similar in women with and without PCOs (11). 

Several studies have shown that the biomarker of vitamin-D, 25-hydroxy vitamin D (25 OH D) is inversely related to body fat percentage, waist circumference, and BMI (body mass index) (8, 12–13). Vitamin D was noted to play an important role in calcium homeostasis. However, vitamin D concentration in women with PCOs and its relationship to insulin resistance and body fat has not yet been fully elucidated. It was reported that women are often insufficient both in calcium and vitamin D (14). 

 It should be mentioned that there have been only few interventional studies about vitamin D supplementation on PCOs in which some characteristics of this syndrome substantially improved but some others have not changed (15-17). Since previous studies have not provided enough evidence to clarify the relationship between vitamin-D and calcium with PCOs and its related factors in adolescence, we conducted this research to evaluate the serum 25 (OH) D and calcium concentrations in high school girls with PCOs in comparison with healthy girls. Also, we compared the serum 25 (OH) D level in PCOs subjects according to obesity and insulin resistance status.

## Methods

**Study subjects: **The first part of this study consisted of 104 high school girls with PCOs and 88 healthy girls, as control subjects aged 16-20 years, selected from another research in screening of PCOs from Jan to March 2012. Laboratory cases and ultrasonographic findings were collected only from case group and the diagnosis of PCOs was based on the Rotterdam criteria (18). Disorders with a similar clinical presentation such as congenital adrenal hyperplasia, Cushing’s syndrome, and androgen-secreting tumors were excluded. 

The control subjects were recruited among healthy girls who were randomly selected from community volunteers. The control subjects had normal menstrual cycles and none of them had clinical evidence of hyperandrogenism. The subjects were not given calcium/vitamin D supplementation calcium/vitamin D during 3 months prior to the study. In order to remove the effect of sunlight on vitamin D synthesis, the experiments were performed in winter. A written informed consent was obtained from each enrolled subject. The research protocol was approved both by the Institutional Review Boards of the Fatemezahra Infertility and Reproductive Health Research Center and the Ethics Committee of Babol University of Medical Sciences. In the second part of this study, we evaluated the relationship between serum vitamin D and calcium and some related factors of PCOs only among the case subjects. The anthropometric indices of the subjects including height, weight, and waist and hip circumference were recorded and the BMI was calculated using the formula [BMI=weight (kg)/ height (2) (cm)]. The participants were divided into four group according to BMI percentile for age and sex based on CDC 2000; underweight (<15 percentile BMI), normal (15-85% BMI percentile), overweight (85-95% BMI percentile) and obese (≥ 95th BMI percentile) (19). Central obesity was considered as waist to hip ratio (WHR) more than 0.88. Hirsutism was evaluated according to the Ferriman-Gallwey (FG) score and was categorized in 4 four groups as normal (FG score<8), mild hirsutism (FG score, range 8-16), moderate (17-25), and severe (>25) (20).

**Biochemical Measurements: **Morning venous samples were obtained after a 12-hour overnight fasting from the subjects between days 2 and 4 of a menstrual cycle or during a spontaneous bleeding episode or progestin-induced menstrual cycle in the PCOs patients. The blood samples were centrifuged and the serum was used for the determination of 25(OH) D, calcium, fasting plasma glucose (FPG) and insulin (FPI), total testosterone, free testosterone, dehydroepiandrosterone sulfate (DHEAS), thyroid-stimulating hormone (TSH), follicle-stimulating hormone (FSH), luteinizing hormone (LH), prolactin (PRL), total cholesterol, HDL and LDL cholesterol and triglycerides. A chemiluminescence immunoassay system (CLIA) was used for the determination of 25(OH) D, calcium, insulin and sex-related hormones (Diasorin, Germany). The serum concentration of glucose, lipid profiles was measured using standard methods (Parsazmoon, Tehran, Iran). Vitamin-D hypovitaminos was defined as serum concentration less than 30ng/ml in two subgroups including insufficient (10-30 ng/ml) and deficient (<10ng/ml). The homeostasis model assessment of insulin resistance (HOMA-IR) was calculated from FPG and FPI according to the equation (21):

[FPG in mg/dl * FPI in mU/ml]/405

**Statistical analysis: **We first compare the serum level of vitamin D and calcium between cases and control using student t-test, The correlations between mean 25(OH) D and calcium with BMI groups, central obesity, HOMA-IR, hirsutism, acne, and polycystic ovary finding at ultrasonography in the PCOs girls were analyzed by Pearson correlation, t-student and chi-square tests considering odds ratio with 95% confidence interval. All statistical analyses were conducted using SPSS Version 18 and a p- value<0.05 was considered statistically significant.

## Results

The mean age of healthy and PCOs girls were 17.6±1.5 and 18.1±11 years, respectively. Also, all PCOs subjects had hypovitaminos D while 5(5.7%) of control group had normal value of serum vitamin D. On the other hand, the mean values of vitamin D were significantly (P=0.04) lower in cases (9.7±4.8) than control group (12.3±11.9). However, calcium level did not differ between the two groups (9.3± 0.3 vs 9.4±0.4). The demographic characteristics and clinical symptoms of PCOs subjects are shown in table 1. 

**Table 1:** Anthropometric and clinical characteristics of PCOs girls

**Features**	**No. (%)**
**BMI(kg/m2)** Underweight Normal Over weight Obese	10 (9.6) 71 (68.3) 16 (15.4) 7 (6.7)
**waist-to-hip ratios (WHR)** ≤0/85 >0/85	74 (71.2) 30 (28.8)
**Acne** Yes No	32 (30.8) 72 (69.2)
**Hirsutism** No (<8) Mild (8-16) Moderate (17-25) Sever ( >25)	22 (21.2) 65 (62.5) 14 (13.5) 3 (2.9)
**Menstruation** Regular Irregular	59 (56.7) 45 (43/3)
**Polycystic ovary at USG** b Positive findings Negative findings	86 (82.7) 18 (17.3)

a As evaluated by Ferriman-Gallwey score

b USG = Ultrasonography

The majority of PCOs subjects had normal weight (68.3%), hirsutism (78.8%) according to the Ferridman-Gallwey score and positive findings of polycystic ovary in the ultrasonography (82.7%). Based on family history of the PCOs girls, obesity (52.9 %), diabetes (38.5 %), irregular menstruation (35.6 %), hirsutism (35.2 %), infertility (22.1%), and alopecia (10.7%) were reported in their family (mothers or first degree relatives). The metabolic variables and parameters of insulin resistance were not significantly different between lean, normal weight, overweight, and obese PCOs patients except triglycerides (table 2).

**Table 2:** The biochemical characteristics of polycystic ovary syndrome (PCOs) subjects in relation to BMI status (n=104

**Variable**	**Lean/ normal weight** ** (n=81)** **Mean±SD**	**Over weight/ obese ** **(n=23)** **Mean±SD**
25(OH) Vit D (ng/ml)	9.6±4.9	9.7±4.2
Calcium(mg/dl)	9.2±0.3	9.3±0.3
Fasting glucose (mg/dl)	85±7	85.5±6.7
Fasting insulin (μU/ml)	11.3±17.6	14.2±6.1
Triglycerides (mg/dl)	94.5±31.9	115.4±52.5 a
Total cholesterol (mg/dl)	149±86	150.3±36.3
LDL (mg/dl)	85±18.6	87.8±28.6
HDL (mg/dl)	46±16	42.1±7.4
Testosterone (ng/l)	0.6±0.7	0.8±0.4
Free testosterone (pg/ml)	3.5±2.3	3.8±1.4
HOMA-IR^b^	2.3±3.3	3±1.4

a p value= 0.02

b HoMA-IR = homeostasis model assessment insulin resistance

**Figure 1:**
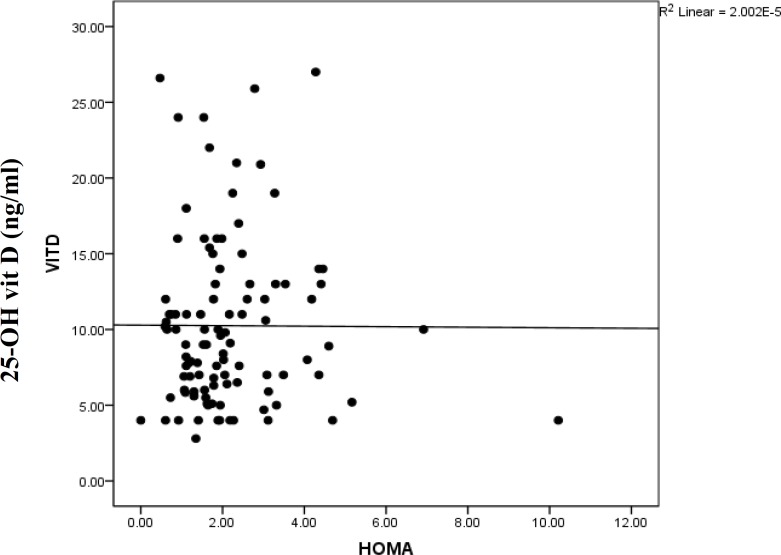
Correlation between 25(OH) vit D and HOMA index (Pearson Correlation) (r= -0.058, P= 0. 56)

As all PCOs subjects had hypovitaminosis D, we compared the differences of some variables like WHR and signs of PCOs in two subgroups including vitamin D insufficiency (10-30 ng/ml) and deficiency (<10ng/ml). However, there was no relationship with the severity of hypovitaminos D and the presence of acne, hirsutism, polycystic ovaries and central obesity (table 3). Based on Pearson's test, no significant correlations were found between 25(OH) D levels with lipid profile, FBS, fasting insulin, and endocrine parameters such as testosterone, free testosterone, FSH, LH, TSH and prolactin. The mean vitamin D among subjects with normal and abnormal HOMA-IR was 9.6±4.6 and 10.5±5.1, respectively. There was no statistically significant correlation found between vitamin-D and HOMA-IR (r=-0.058, P=0.56) (Fig 1).

**Table 3:** The relationship between the severities of hypovitaminosis D with some clinical symptoms of PCOs

**Variable**	**Vit D Deficiency** **(<10)** **N (%)**	**Vit D Insufficiency** **(10-30)** **N (%)**	**OR (CI 95%), p value**
**Central Obesity** No ayes	34 (45.9) 13 (43.3)	40 (54.1) 17 (56.7)	1 0.9(0.3-2.1), p=0.491
**Acne** no yes	29 (44.6) 18 (46.2)	36 (55.4) 21 (53.8)	1 1.0(0.4-2.3), p=0.520
**Hirsutism** no yes^b^	9 (40.9) 38 (46.3)	13 (59.1) 44 (53.7)	1 1.2(0.4-3.2), p= 0.417
**Polycystic ovary** no at USG^c^ yes	7 (38.9) 50 (58.1)	11 (61.1) 36 (41.9)	1 0.4(0.1-1.2), p=0.109

a Central Obesity: waist-to-hip ratios (WHR) ≥ 0. 85

b Ferriman-Gallwey score ≥ 8

c USG = Ultrasonography

## Discussion

The present study was designed to compare the level of 25 (OH) D and calcium according to some features and characteristics of PCOs in young girls who were not studied in details with the induction of PCOs complication. According to the result of this study, all PCOs subjects had hypovitaminos D and the mean value of vitamin D was significantly lower than non-PCOs girls. However, most of our study subjects (>95%) had this problem. In other studies, thay had reported different values. Vitamin D deficiency imbalance varied from 32% to 52% which was related to race, seasons and other features. (22-25) On the other hand, the low levels of 25(OH) D in PCOs girls in our study were in concordance with some other studies in adults (26, 27). In non-PCOs cohorts including immigrant and non-immigrants, subjects, hypovitaminos D in immigrant adolescent girls had significantly increased (28). In contrast, two other studies showed an increase level of 25 (OH) D in PCOs subjects. It seems that this difference may be due to the age of the samples. The study findings indicated no significant difference in calcium level between patients and controls. Our findings are consistent with earlier studies. (17, 29- 31). Based on the effect of obesity on some clinical aspects, the complications of PCOs were reported in other studies (32-35). We compared the mean value of 25 (OH) D and calcium, glucose and lipid profile between the two groups on the basis of percentile of BMI. 

As shown in table 2, there was no significant difference in vitamin D and calcium. These data are in agreement with the results reported by Mahmody et al. (30). But several studies in adolescents with PCOs mentioned the relationship between 25 (OH) D levels and BMI (26, 29, 34, 35) confirmed that the lower value of vitamin D may be due to obesity, not PCOs (29). The distribution of normal BMI was 68.3% in our study subjects in which may be one of the causes of these differences.

There was no association between the severity of hypovitaminos D and symptoms of PCOs. Also, there was no significant correlation between central obesity and severity of hypovitaminos. This pattern was in contrast with the finding of Wehr et al. (26). 

We found no significant difference in fasting glucose and insulin level and HOMA IR in PCOs girls according to BMI. These findings are in agreement with previous reports (36) except glucose (30, 37). But in contast with the study by Hahn et al. (29). In the evaluation of the lipid profile in PCOs girls, according to BMI, we found no significant difference in the cholesterol, LDL and HDL level. The only statistical difference that has been observed was related to triglycerides in different BMI, so the mean serum triglyceride level in the obese, overweight groups was higher than the lean, normal weight groups. The results were similar to some studies. (29, 37) but were in contrast with the others (38). We found no statistically significant differences between vitamin D and HOMA-IR. Also, the data demonstrate a lack of significant association between 25 (OH) D levels and HOMA IR in women with PCOs in the 20-29 years and 30-40 years groups, respectively (39). However, the results of our studies were in contrast with previous studies (29, 26). 25 (OH) D correlated negatively with HOMA IR. Also, age is an important risk factor for developing metabolic disorders. According to the previous longitudinal study, BMI, triglyceride, cholesterol and LDL levels increased in women with PCOs over time (39, 40).

 Despite the previous reports, (26) we did not find a correlation between vitamin D with FBS, fasting insulin, triglycerides and HDL. Obesity is seen in 40% to 50% of women with PCOs. This obesity is usually of the android type, with an increased WHR (41, 42). In this research, most cases were normal in weight and WHR≤0.88. Clinical and biochemical consequences of androgen excesses are the major characteristics of women with PCOs. An age-related decrease in androgen secretion occurs in women with PCOs, as it does in normal women. Ovarian steroid secretion starts to decline as early as age 30 (43). 

Our observations on signs and symptoms of PCOs are in line with a previous study (38, 44). Also, in one study, it was reported that older women with PCOs have a lower Ferriman-Gallwey score (37). Regarding family history, we observed that more than half of PCOs girls had family history of obesity, the rates of family history of hirsutism, diabetes and menstrual disorders in our study were similar to other reports (45-48). Our study has several limitations that should be noted. First, the sample size was small. Therefore, the samples were not normally distributed in terms of BMI. Second, the subjects in the study had a specific age group. Third, the metabolic, endocrine, and demographic factors of control group were not evaluated for comparison but were assessed only in terms of vitamin D and calcium.

In conclusion, although all PCOs high school girls had hypovitamionos D, there was no correlation between vitamin D deficiency and its severity with some features and complications of PCOs including obesity, insulin resistance in this age group. 

## Notes

**Funding:** This study was supported by a grant (No. 8928622) from Babol University of Medical Sciences, Iran.

## Notes

**Conflict** **of Interest:** The authors declare no conflict of interests.
